# Mutations in the *PIGW* gene associated with hyperphosphatasia and mental retardation syndrome: a case report

**DOI:** 10.1186/s12887-019-1440-8

**Published:** 2019-02-27

**Authors:** Li’na Fu, Yan Liu, Yu Chen, Yi Yuan, Wei Wei

**Affiliations:** 1 Department of Pediatrics, Affiliated Tongji Hospital of Tongji Medical College of Huazhong University of Science and Technology, Wuhan, 430000 China; 2 Kangso Medical Inspection, Beijing, China

**Keywords:** *PIGW*, Epilepsy, Delayed cognitive development, Alkaline phosphatase

## Abstract

**Background:**

Mutations in the *PIGV*, *PIGO*, *PIGL*, *PIGY*, *PGAP2*, *PGAP3,* and *PIGW* genes have recently been reported to cause hyperphosphatasia accompanied by mental retardation syndrome (HPMRS); the latter is an autosomal-recessive neurological disorder typically characterised by recurrent seizures, intellectual disability, and distinct facial features. Here, we report an extremely rare case of a Chinese boy with compound heterozygous PIGW mutations who suffers from severe pneumonia, mental retardation, and epilepsy.

**Case presentation:**

A 70-day-old boy presented with fever and cough over 20 days in duration at the time of admission. At the age of 6 months, unusual facial features were apparent, and seizures were clinically observed, accompanied by obvious cognitive delay. Next-generation sequencing identified novel PIGW c.178G > A and c.462A > T mutations, confirmed by Sanger sequencing.

**Conclusions:**

Mutations in the *PIGW* gene in infants can cause various symptoms and multiple anomalies. Next-generation sequencing efficiently detects such mutations. The compound *PIGW* mutations that we describe expand the genotype/phenotype spectrum of HPMRS and may aid in clinical treatment.

## Background

Glycosylphosphatidylinositol (GPI) is a cell surface glycolipid that anchors over 150 proteins to the cell membrane; these proteins include enzymes, receptors, and adhesion molecules that are involved in signal transduction [1]. At least 26 genes are involved in the synthesis and remodelling of GPI-anchored proteins [2], and these play indispensable roles in embryonic development. Although complete GPI deficiency triggers embryonic death [3], postnatal GPI deficiency is not usually fatal. Of the 26 genes, 22 are termed *PIG* (phosphatidyl inositol glycan) genes, and the remaining 4 are *GPAP* (post-GPI attachment to protein) genes. *PIG* genes are involved in synthesis of the GPI anchor (and a precursor) in the endoplasmic reticulum (ER); GPI becomes attached to newly produced proteins with appropriate signal sequences. *PGAP* genes modify GPI in the ER and Golgi [4]. Patients with inherited GPI deficiencies (IGDs) usually present with cognitive delay, epilepsy, multiple organ anomalies, coarse facial features, such as a wide nasal bridge and tent-shaped lips, and an inguinal hernia.

Hyperphosphatasia with mental retardation syndrome (HPMRS), also termed Mabry syndrome, is caused by IGD and is inherited in an autosomal-recessive manner. The typical features include intellectual disability, distinctive facial features, epilepsy, hyperphosphatasia, and multiple organ anomalies. Disease severity is associated with the extent of the genetic defect and the resulting impairment of synthesis pathways [5]. Mutations in *PIG* genes such as *PIGV*, *PIGO*, *PIGL*, *PIGY*, *PGAP2*, *PGAP3,* and *PIGW* cause HPMRS. Symptom heterogeneity is widespread, according to a recent review of HPMRS cases from Europe and America; the clinical manifestations and mutations in *PIGV*, *PIGO*, and *PGAP2* were summarised [6]. The clinical features vary even when the mutations occur in the same gene. The *PIGW* gene is responsible for the third step in GPI synthesis, acylation of the inositol ring [7]. *PIGW* mutations causing HPMRS are very rare. The first case was a Japanese individual, reported in 2013, who exhibited compound heterozygous mutations [5]. In 2016, a German report described two patients with the same homozygous mutation in *PIGW* [4]. Here, we report a case of HPMRS associated with compound heterozygous mutations in *PIGW*; the male patient presented with pneumonia, developmental delay, epilepsy, and coarse facial features.

## Case presentation

A male infant was spontaneously delivered after 39 weeks of gestation and was diagnosed with pneumonia at the age of 15 days. He was the second child of a non-consanguineous family. His elder brother had died at the age of 7 months because of recurrent pulmonary infection; that child had exhibited obvious developmental delay and could not raise the head or turn over at the time of his death. The patient was first admitted to our hospital at 70 days of age after 20 days of intermittent fever and cough; his body weight was 5.6 kg. Coarse facial features (a wide nasal bridge; tent-shaped lips; and high, narrow palatine arches) were noted; he nodded his head during breathing and exhibited three signs of depression. An umbilical hernia and bilateral indirect inguinal hernias were evident, but were amenable to repair. Prior to sleeping, his eyes often blinked and turned upwards, but these features resolved spontaneously. Lower extremity muscle strength and tone were normal.

**Fig. 1:**
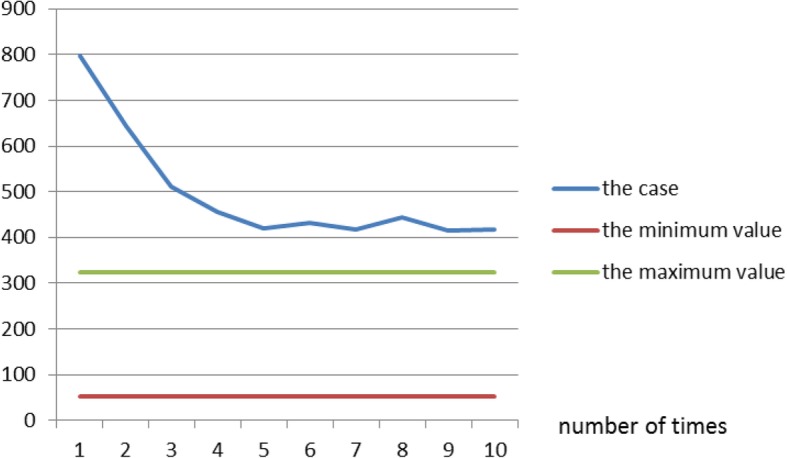
The serum ALP level in our case, and the normal range

The serum ALP level (414–798 U/L) after admission was higher than normal (Fig. 1). Renal function, assessed by measuring the creatinine clearance rate, and blood myocardial enzyme levels were normal. Serological tests ruled out cytomegalovirus (CMV), hepatitis B and C, syphilis, rubella, toxoplasmosis, Epstein–Barr virus, and human immunodeficiency virus infections. Blood and sputum cultures were negative. The levels of blood immunoglobulins and lymphocyte subsets were normal, as was the cerebrospinal fluid analysis. Video electroencephalography (EEG) performed during the interictal period revealed many sharp waves, accompanied by sharp, slow discharges in both the temporal and frontal regions, and partial-onset epileptic seizures, which were treated by administration of oxcarbazepine. Cardiac ultrasonography was normal. Chest computed tomography revealed bilateral lung infections, and head magnetic resonance imaging showed widening of the subarachnoid space in both frontotemporal regions.

The patient underwent endotracheal intubation, assisted ventilation, and anti-infection and anti-epileptic treatments. His body temperature fell to normal after 2 weeks. However, the seizures could not be completely controlled. Also, the patient could not raise his head or trace moving objects with his eyes, even at the age of 5 months. His body weight increased very slowly, reaching 6 kg at the age of 9 months. Severe psychomotor retardation was evident; only at the age of 9 months was he able to turn over, sit, and to respond to his name.

Genetic analysis.

Next-generation sequencing of the whole exome was performed when the patient was 5 months of age to seek potential genetic defects. Genomic DNA of the patient and his parents were extracted from peripheral blood using a Qiagen FlexiGene DNA kit (Qiagen, Germany). A microarray chip was used to capture the entire exome, followed by sequencing of all exons, together with the flanking 10-bp regions of introns, on an Illumina NovaSeq 6000 platform. Clean reads were aligned against the human assembly GRCh37/hg19 using the Burrows–Wheeler Aligner. The mapping rate of the target regions was 99.9%, and the average depth was 124x. Polymorphisms were removed by reference to their population frequencies by searching for such mutations in genetic disease databases including OMIM (http://omim.org), HGMD (http://www.hgmd.cf.ac.uk/ac/index.php), ClinVar (https:www.ncbi.nlm.nih.gov/clinvar), the database of the 1000 Genomes Project (http://www.1000genomes.org/), ESP6500 (http://evs.gs.washington.edu/EVS/), and ExAC (http://exac.broadinstitute.org). The effects of mutations on protein function were predicted with the aid of PolyPhen 2 (http://genetics.bwh.harvard.edu/pph2/), SIFT (http://sift.jcvi.org), and Mutation Tester (http://www.mutationtaster.org), followed by further assessment in relation to clinical characteristics.

**Fig. 2:**
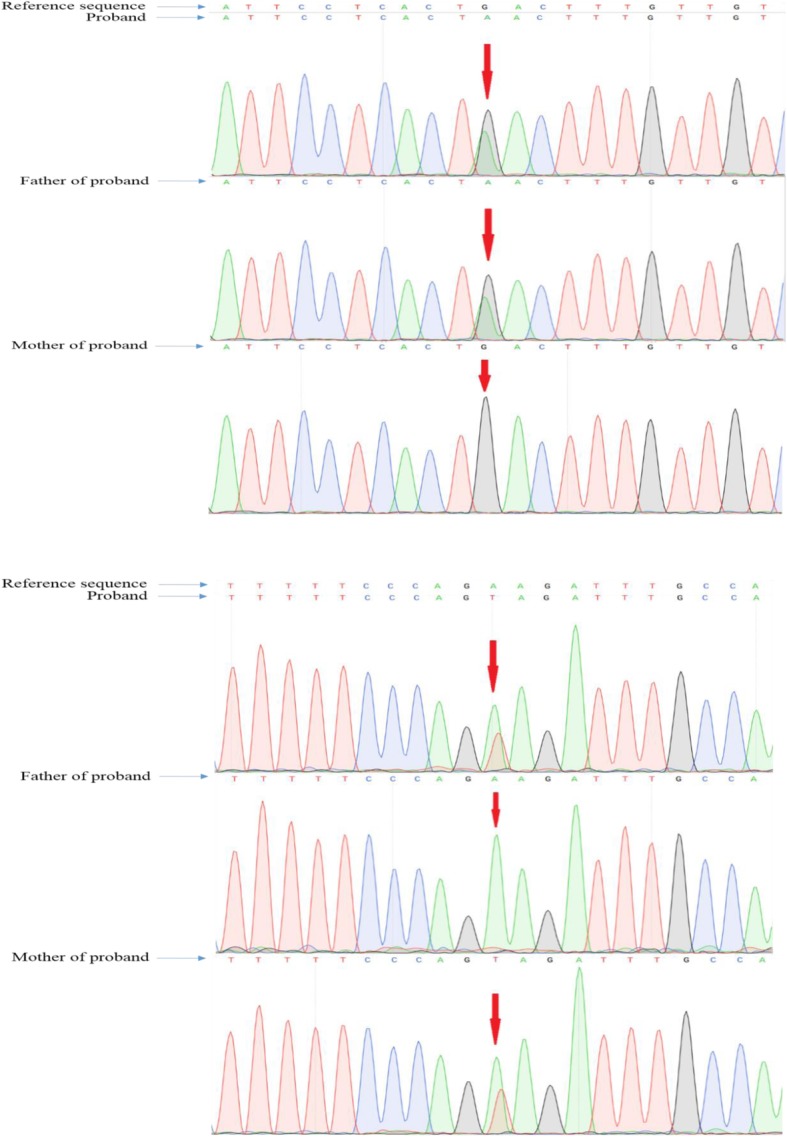
Sequencing of the *PIGW* gene. The arrows indicate the positions of mutations. **(A)** DNA sequencing profile showing the paternal mutation, c.178G > A, in exon 2 of *PIGW*. (**B)** DNA sequencing profile showing the maternal mutation, c.462A > T, in exon 2 of *PIGW*

**Fig. 3:**
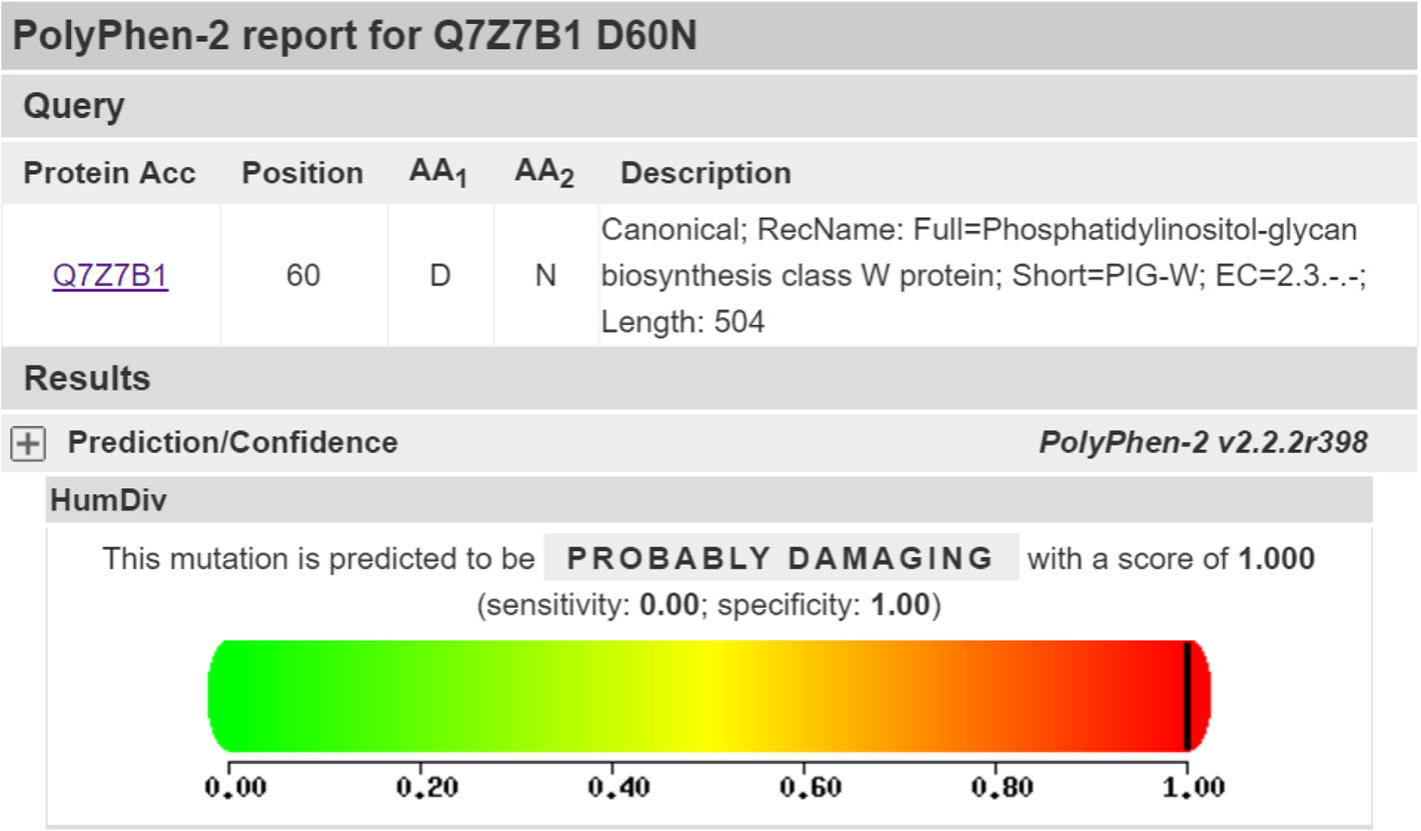
PolyPhen 2 prediction of the loss of function caused by the missense mutation c.178G > A (p.Asp60Asn) inherited from the father

**Fig. 4:**
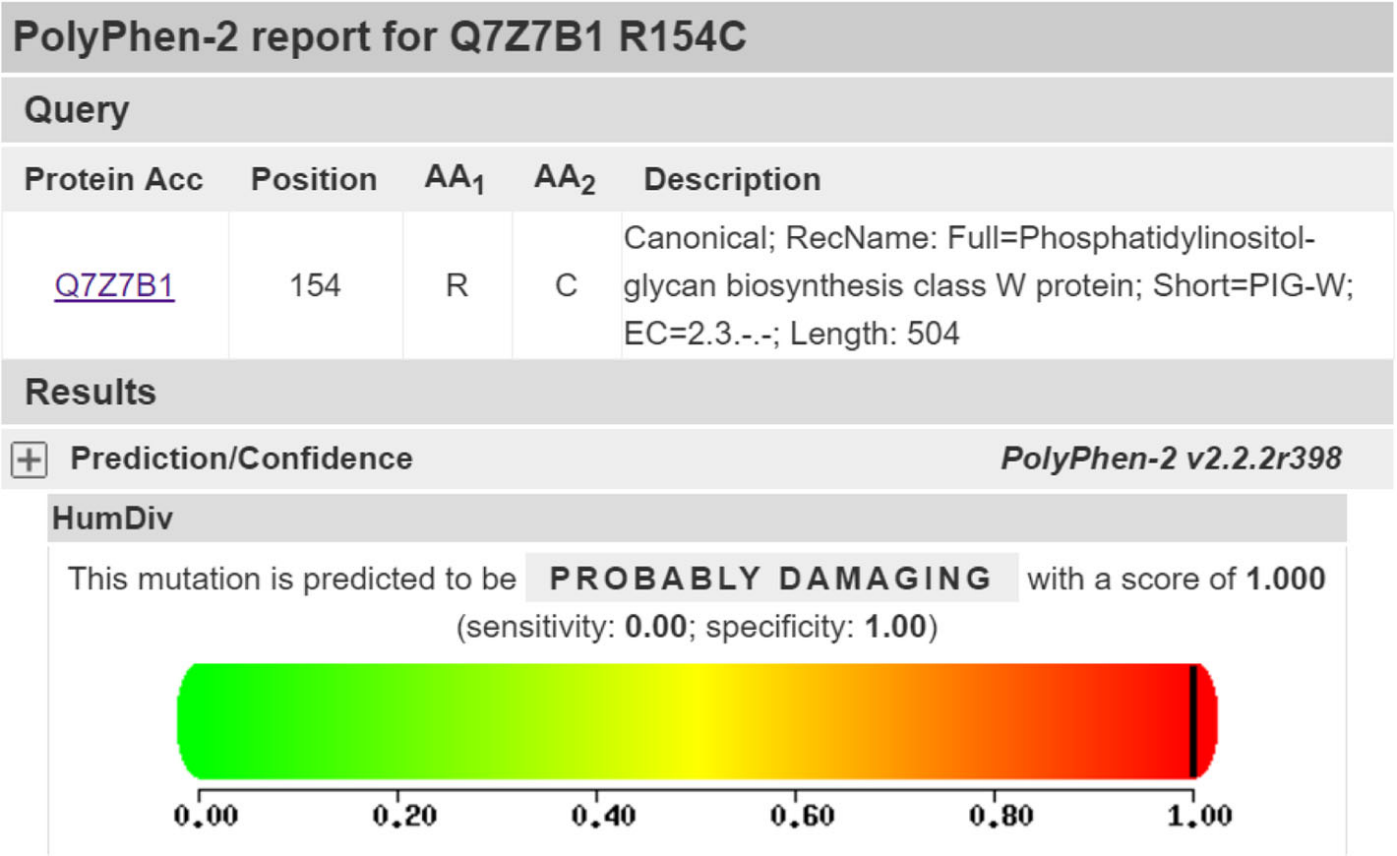
PolyPhen 2 prediction of loss of function caused by the missense mutation c.462A > T (p.Arg154Ser) inherited from the mother

The patient had inherited compound heterozygous mutations in *PIGW*, c.178G > A (p.Asp60Asn) and c.462A > T (p.Arg154Ser), from his father and mother, respectively (Fig. 2). Both variants are very rare, as shown by the “1,000 Human Genomes” database. PolyPhen 2 predicted that both missense mutations compromised protein function (Figs. 3 and 4). No other relevant mutations were found.

## Discussion and conclusions

Typically, GPI deficiencies cause intellectual disability, seizures, and facial dysmorphisms. However, the symptoms vary greatly among patients. The phenotypes associated with different mutations also vary greatly. Transcriptional changes caused by mutations in *PIGV* and *PIGO* reduce membrane protein stability and/or impair enzymatic function. The synthesis of GPI-anchored proteins is also affected by decreases in the level of the GPI substrate [8, 9]. *PGAP2* is associated with remodelling of GPI-anchored fatty acids; under normal conditions, these stabilise GPI-anchored proteins and the cell membrane [10]. Proteins encoded by *PIGW* and *GPI* catalyse inositol acylation, an early step in GPI synthesis. Cells with *PIGW* defects accumulate intermediate products deficient in inositol acylation. The four known gene mutations cause various clinical forms of HPMRS. A 2014 European study reviewed the clinical data of 16 HPMRS cases caused by mutations in *PIGV*. The most common symptoms were hyperphosphatasia, epileptic seizures, abnormal facial features, and severe retardation [11]; additional symptoms included lesions of the bladder, ureters, and kidneys and anorectal malformations. *PIGV* mutations were also associated with palatine clefts and heart disease [11]. An earlier 2012 study revealed that compound heterozygous *PIGO* mutations also triggered HPMRS [8]. The clinical manifestations included abnormal facial features, moderate to severe developmental delays, hypoplasia, congenital absence of the terminal toes, and hyperphosphatasia with or without urinary system/heart malformations. Compared to *PIGV* mutations, *PIGO* mutations cause more severe developmental retardation [8]. Other studies also found that the clinical features associated with *PGAP2* mutations included severe HPMRS and mild cognitive delay [10].

However, hyperphosphatasia has been reported only in cases with IGDs caused by mutations in *PIGV*, *PIGO*, *PGAP2,* and *PIGW* [12]. Murakami et al. proposed that this reflected high-level alkaline phosphatase (ALP) secretion. Mutations affecting early steps in GPI biosynthesis would be associated with normal plasma ALP levels because such mutations trigger only intracellular degradation of the precursor ALP protein; mutations affecting later biosynthetic steps would trigger high levels of plasma ALP [13]. However, Hogrebe et al. reported a case of GPI deficiency lacking hyperphosphatasia and caused by a new *PIGW* mutation discovered in Germany [4]. Two cousins carried the *PIGW* homozygous mutation c.460A > G (p.R154G). Their symptoms differed remarkably from those of patients with other *PIGW* mutations. A transfection experiment strongly supported the idea that enzymatic activity was affected by the mutation.

One Japanese case featured compound heterozygous mutations of *PIGW*: c.211A > C (p.Thr71Pro) and c.499A > G (p.Met167Val) [5]. The patient presented with developmental delay in early infancy, mildly abnormal facial features (a wide nasal bridge and a tent-like upper lip), and an inguinal hernia. The patient was initially diagnosed with West syndrome because interictal EEG revealed a high-amplitude hyperrhythmic pattern. The laboratory data were normal except for a considerable elevation in the serum ALP level.

Similarly, our patient presented with retarded development, abnormal facial features (a wide nasal bridge, high and narrow palatine arches, and a tent-shaped upper lip), an inguinal hernia, hyperphosphatasia, and partial-onset epileptic seizures treated with oxcarbazepine. However, anti-epileptic treatment was not fully effective. Next- generation sequencing revealed compound heterozygous mutations in PIGW, c.178G > A and c.462A > T, inherited from a parent.

Notably, apart from the abovementioned, common symptoms of HPMRS, both the patient and his elder brother had suffered from recurrent pulmonary infections. The patient’s brother had died of an infection at the age of 7 months. The major respiratory symptoms of our patient were cough and fever. Chest CT revealed severe bilateral lung infections; the patient required endotracheal intubation, assisted ventilation, and antibiotics. It remains unknown whether HPMRS caused by a defective *PIGW* gene is associated with pneumonia. It is unclear whether the recurrent pulmonary infections in the two brothers were caused by the *PIGW* gene defect or by feeding intolerance attributable to retarded development. In 2015, an Australian study reported a patient with *PIGY* mutations who exhibited similar dysmorphic features: brachyphalangy, proximal limb shortening, contractures, and left hip dysplasia [14]. Her development regressed at the age of 5 months, and her vision was so poor that she remained largely unresponsive. She died at 7 months of age secondary to an operation seeking to establish aspiration [14]. Although the report is unclear, we suggest that the cause of death was aspiration pneumonia. Although no other cases of recurrent pneumonia have been reported, we suggest that recurrent pulmonary infection may be a new HPMRS phenotype. Further work is needed.

HPMRS caused by *PIGW* mutations is very rare. Our case presented with pneumonia, psychomotor delay, epilepsy, and coarse facial features. Multiple anomalies are evident in infants with *PIGW* gene mutations. Next-generation sequencing efficiently identifies relevant mutations. Further comprehensive studies are needed to explore how known mutations affect GPI biosynthesis and protein anchoring in various tissues. In vitro transfections of plasmids carrying mutated genes will increase our understanding of how mutations affect protein function.

## Abbreviations

GPI: Glycosylphosphatidylinositol; IGD: Inherited GPI deficiency
HPMRS: Hyperphosphatasia with mental retardation syndrome
